# Improving the therapeutic profile of MSCs: Cytokine priming reduces donor-dependent heterogeneity and enhances their immunomodulatory capacity

**DOI:** 10.3389/fimmu.2025.1473788

**Published:** 2025-02-17

**Authors:** Jaris Valencia, Rosa M. Yáñez, Sandra Muntión, María Fernández-García, Jorge Diego Martín-Rufino, Agustín G. Zapata, Juan A. Bueren, Ángeles Vicente, Fermín Sánchez-Guijo

**Affiliations:** ^1^ Department of Cell Biology, School of Medicine, Complutense University of Madrid, Madrid, Spain; ^2^ Heath Research Institute Hospital Clínico San Carlos (IdISSC), Madrid, Spain; ^3^ RICORS TERAV, Instituto de Salud Carlos III (ISCIII), Madrid, Spain; ^4^ Hematopoietic Innovative Therapies Division, Centro de Investigaciones Energéticas, Medioambientales y Tecnológicas (CIEMAT) and Centro de Investigación Biomédica en Red de Enfermedades Raras (CIBERER), Madrid, Spain; ^5^ Heath Research Institute-Fundación Jiménez Díaz (IIS-FJD), Madrid, Spain; ^6^ Department of Medicine, University of Salamanca and Cell Therapy Area and Hematology Department, IBSAL-University Hospital of Salamanca, Salamanca, Spain; ^7^ Regenerative Medicine and Cellular Therapy Network Center of Castilla y León, Salamanca, Spain; ^8^ Division of Hematology/Oncology, Boston Children’s Hospital and Department of Pediatric Oncology, Dana-Farber Cancer Institute, Harvard Medical School, Boston, MA, United States; ^9^ Broad Institute of MIT and Harvard, Cambridge, MA, United States; ^10^ Department of Cell Biology, Faculty of Biology, Complutense University of Madrid, Madrid, Spain; ^11^ Heath Research Institute Hospital 12 de Octubre (I+12), Madrid, Spain

**Keywords:** mesenchymal stem cells, mesenchymal stromal cells, MSCs, cytokines, priming, heterogeneity, immunomodulation, innate immune system

## Abstract

**Introduction:**

MSCs exhibit regenerative, anti-inflammatory and immunomodulatory properties due to the large amount of cytokines, chemokines and growth factors they secrete. MSCs have been extensively evaluated in clinical trials, however, in some cases their therapeutic effects are variable. Therefore, strategies to improve their therapeutic potential, such as preconditioning with proinflammatory factors, have been proposed. Several priming approaches have provided non-conclusive results, and the duration of priming effects on MSC properties or their response to a second inflammatory stimulus have not been fully addressed.

**Methods:**

We have investigated the impact of triple cytokine priming in MSCs on their characterization and viability, their transcriptomic profile, the functionality of innate and acquired immune cells, as well as the maintenance of the response to priming over time, their subsequent responsiveness to a second inflammatory stimulus.

**Results:**

Priming MSCs with proinflammatory cytokines (CK-MSCs) do not modify the differentiation capacity of MSCs, nor their immunophenotype and viability. Moreover, cytokine priming enhances the anti-inflammatory and immunomodulatory properties of MSCs against NK and dendritic cells, while maintaining the same T cell immunomodulatory capacity as unstimulated MSCs. Thus, they decrease T-lymphocytes and NK cell proliferation, inhibit the differentiation and allostimulatory capacity of dendritic cells and promote the differentiation of monocytes with an immunosuppressive profile. In addition, we have shown for the first time that proinflammatory priming reduces the variability between different donors and MSC origins. Finally, the effect on CK-MSC is maintained over time and even after a secondary inflammatory stimulus.

**Conclusions:**

Cytokine-priming improves the therapeutic potential of MSCs and reduces inter-donor variability.

## Introduction

Mesenchymal stem/stromal cells (MSCs) are present along the connective tissue of the body, where they play an important role in tissue homeostasis, remodeling and repair. They have been widely assessed in different clinical applications based on their anti-inflammatory, immunomodulatory and regenerative potential (1–3). In this regard, they have capacity to differentiate into cells of the mesodermal lineages and to migrate to inflammatory tissues where they induce local repair by the secretion of many growth factors and by reducing inflammation (4, 5). Moreover, MSCs modulate the immune system through the secretion of important bioactive molecules (2, 6) and are hypoimmunogenic themselves making them suitable for allogeneic transplantation. Therefore, MSCs are very attractive candidate for the treatment of inflammatory, immune-mediated, and degenerative diseases treatment (7), although phase III clinical trials have provided variable results and only a few MSC-based products have reached the market (8).

It has been shown that MSCs are able to modulate innate and adaptive immune responses. Thus, they can suppress the activation of natural killer (NKs) cells, the differentiation and maturation of dendritic cells (DCs) and promote macrophage polarization toward an anti-inflammatory phenotype. In addition, they inhibit the proliferation and function of T and B lymphocytes while induce the generation of T regulatory cells (Tregs) (9).

Initially it was thought that MSCs display baseline immunomodulatory ability, but many studies have shown that they require some stimuli, including inflammation, three-dimensional interactions with extracellular matrix, hypoxia or interactions with different cells of the immune system (10–12). Under inflammatory conditions, MSCs are “licensed/activated/pre-stimulated” and show and increase in proliferation and survival, and acquire enhanced immunomodulatory ability (13), producing large amounts of factors classically related with immunosuppression, such as indoleamine-2,3-dioxygenase (IDO), transforming growth factor- β1 (TGF- β1)-, prostaglandin-E2 (PGE2), interleukin-6 (IL-6), interleukin -10 (IL-10), HLA-G or nitric oxide (NO), among others. Interestingly, MSCs can become immunosuppressive under strong inflammation, while weak inflammation enhances their immune responses, leading to the concept of bidirectional interaction between MSCs and inflammation (14).

On this immunomodulatory basis, growing evidence suggests that MSC administration may be a promising therapy for the treatment of inflammation. MSCs have obtained clinical responses in various inflammatory or immune-mediated diseases like graft-versus-host disease (GvHD) (15, 16), Crohn´s disease (17), multiple sclerosis (18) or rheumatoid arthritis (19) although, as it has been mentioned, few phase III clinical trials using MSCs have obtained significant results leading to regulatory approvals (8). If we take the role of MSC for steroid-refractory acute GVHD as an example (since it is the clinical indication in which MSC have been more deeply assessed), a deep analysis of these phase III trials made (20) showed that some of them had pitfalls either in their design, in the definition of primary endpoints and/or in the target population, which could be optimized. For this reason, more recent Phase III trials are already showing more clearly a significant advantage of MSC in this setting (21, 22), leading to FDA approval in December 2024 of the first BM-MSC-based cell product for the treatment of refractory GVHD (23).

In this context, different strategies have been developed to improve the therapeutic potential of MSCs including increase of their survival, migration and homing to damaged tissues, and improvement their capacity to secrete different immunomodulatory factors (3), such as expansion under hypoxic conditions, 3D cultures with biomaterials or priming with proinflammatory cytokines (CKs) (24).

Nevertheless, many aspects remain to be answered to improve the therapeutic potential of MSCs by using pre-conditioning approaches, including how long the effects of priming on the immunomodulatory capacity of MSCs last, or whether these effects are transient or sustained over a period of time, or after exposure to a second stimulus, mimicking what would happen when these cells are infused into a patient with an inflammatory disease. Another important issue is the impact of the MSCs heterogeneity due to the different donors or cell sources used (25). Accordingly, in this report we have investigated the effects of MSCs priming with a proinflammatory CK cocktail consisting of IFNγ, TNFα, and IL-1β on different aspects of MSC biology, particularly on their immunomodulatory capacity.

## Materials and methods

### Human samples

Bone marrow (BM) samples were obtained from 9 healthy donors (4 females/5 males) with a median age of 30 years (range: 18–55 years) by iliac crest aspiration in sterile conditions under local anesthesia.

Adipose tissue (AT) samples were obtained by lipoaspiration from 11 healthy donor (7 females/4males) with a mean age of 48.6 years. Five samples were purchased from Caltag MedSystem (Buckingham, UK) after informed consent in accordance with the Helsinki Declaration and all donors signed a specific informed consent form approved by the Ethics Committee of the University Hospital of Salamanca and by the Ethics Committee of Fundación Jiménez Díaz Hospital.

Heparinized peripheral blood samples from healthy donors were obtained after signed informed consent and in compliance with the Helsinki Declaration, from the Centro de Transfusión de la Comunidad de Madrid and the Centro de Hemodonación de Castilla y León under their respective Institutional Review Boards approval.

### MSC culture

To obtain BM-MSCs, BM mononuclear cells were obtained by density-gradient centrifugation with Ficoll-Paque (1.077 g/ml; GE Health-care BioSciences, AB, Uppsala, Sweden), and seeded at 1×10^6^ cells/cm^2^ on culture flasks with Dulbecco’s Modified Eagle Medium (DMEM) supplemented with 10% platelet lysate (Cook Medical, IN, USA) and 1% penicillin/streptomycin (all from Gibco, Thermo Fisher), referred as to DMEM completed medium, as previously described (26).

To obtain AT-MSCs, the lipoaspirate was disaggregated and digested with collagenase A (Serva, Germany) at a final concentration of 2 mg/ml for 4 h at 37°C, filtered through 100-μm nylon filters (BD Bioscience, USA) and centrifuged for 10 min. Cells were seeded at a concentration of 10,000 cells/cm^2^ in culture flasks (Corning, USA) with Minimum Essential Medium α (α-MEM; Gibco/Life Technologies/Thermo Fisher Scientific, Waltham, USA) supplemented with 5% platelet lysate (Cook Medical, IN, USA), 1% penicillin/streptomycin (Gibco/Life Technologies/Thermo Fisher Scientific, Waltham, USA), and 1 ng/ml human basic fibroblast growth factor (bFGF; Peprotech, NJ, USA) and cultured at 37°C.

For MSC expansion, the cells were cultured in completed medium and fed by complete replacement of the medium every 2–4 days. Adherent cells were serially passaged using 0.25% trypsin/EDTA (Sigma Aldrich, USA) upon reaching 70%–90% of confluence. MSCs between 3rd-6th passages were used for all the experiments.

For MSC pre-stimulation, 5x10^5^ cells were seeded and after 24h, MSC were stimulated with IFN-γ (20ng/ml), TNF-α (10ng/ml) and IL-1β (20ng/ml) (all from PeproTech, Rocky Hill, USA) during 24h and will be referred to as cytokine (CK)-MSCs.

### Differentiation assays of MSCs

For osteogenic differentiation, basal and pre-stimulated AT-MSCs were maintained for 10 days with osteogenic differentiation medium (Miltenyi Biotec, Bergisch Gladbach, Germany) and then stained with nitro-tetrazolium blue chloride/5-bromo-4-chloro-3-indolyl phosphate (NBT/BCIP).

For adipogenic differentiation, cells were kept for 21 days in culture with adipogenic differentiation medium (Miltenyi Biotec, Bergisch Gladbach, Germany) and then evaluated for the presence of lipid vacuoles stained with oil red staining.

All media were removed twice a week during differentiation. Images were obtained on an Olympus BX41 microscope (Olympus, Tokyo, Japan) at 10X magnification.

### RNA-Seq studies

#### RNA extraction

RNA from 1x10^6^ AT-MSCs under basal or pre-stimulated conditions was extracted using the QIAGEN Rneasy plus mini kit (Qiagen, Hilden, Germany) according to the manufacturer’s instructions. RNA samples were quantified using the Qubit 2.0 fluorimeter (Life Technologies, Carlsbad, USA) and RNA integrity was checked using the 4200 TapeStation instrument (Agilent Technologies, Palo Alto, USA). All samples had ≥200 ng/mL, and RNA integrity number (RIN) ≥ 8.0 and then subjected to RNA-Seq.

#### Library preparation

The library was prepared using the NEBNext Ultra RNA Library Prep Kit for Illumina (NEB, Ipswich, USA). Briefly, mRNA from each sample was enriched with oligo primers (dt). Then, all of them were fragmented for 15min at 94°C, and complementary DNA (cDNA) strands were synthesized. The ends of these cDNAs were repaired and adenylated at the 3’ end, adaptors were ligated, indexes were added, and the library was enriched by PCR. The cDNA library was validated on an Agilent TapeStation and quantified using the Qubit 2.0 fluorimeter and by quantitative PCR (KAPA Biosystem, Wilmington, USA). Finally, it was sequenced on a paired end flow cell on the Illumina HiSeq sequencer with a depth of 150bases pairs (2x150bp) and an average of 34 million paired reads per sample.

#### Mass sequencing

The cDNA library of pre-stimulated and control MSCs was sequenced following the RNA-Seq protocols. The raw read data in “bcl” format were converted to “. fastq” files and de-multiplexed using the bcl2fastq2.17 software. Quality control was performed using the bioinformatics tools FastQC v0.11.8 and MultiQC v1.7. Sequencing adapters and low-quality reads (stringency 5) were removed with the Trim Galore package. The Salmon pseudo-aligner was used to calculate the expression levels of the transcripts in each sample using the human transcriptome hg38 as a reference. The amount of each transcript was imported into the differential expression analysis package DESeq in the statistical software R v3.6.0.

#### Functional enrichment analysis

To understand the biological impact of gene expression changes, a functional enrichment analysis of differentially expressed genes was performed using the PATHER tool and Gene Ontology (GO). Biological processes were selected as significantly enriched by p-value adjusted by FDR<0.05.

### Gene expression analysis by qRT-PCR

High-capacity cDNA Reverse transcription kit (Applied Biosystems. Thermo Fisher Scientific) was used for the synthesis of cDNA of BM-MSCs according to manufacturer’s instructions. Quantitative real-time PCR (qRT-PCR) was performed using specific predesigned TaqMan Gene expression assays for different genes (Applied Biosystems) (**Supplementary Table S1**). All PCR reactions were set in duplicates using the TaqMan Gene Expression Master Mix (Applied Biosystems). The amplification and detection were performed using a 7900HT Fast Real-time PCR System (Centro de Genómica, Complutense University of Madrid). ΔCT method was employed using *GNB2L1* as reference gene to normalize gene expression.

### Immunomodulatory profile of MSCs

Control and CK-MSCs from adipose tissue were trypsinized and labelled for 15 min in the dark with the following combination of monoclonal antibodies: CD54- APC/Fire™750, CD90- PE-Cy7 and CD105 Brillant violent 421™ from BioLegend, and CD106-PE, CD273- PerCP-Cy5.5 and CD274- Brillant blue from BD Bioscience. Dead cells were excluded by 7-amino-actinomycin D (7AAD) (eBiosciences) staining. Cells were acquired in a FACSCanto II flow cytometer (BD Bioscience) and analysed using Infinicyt 1.8 software (Cytognos-BD, Salamanca, Spain).

### PBMC, NK-cell and monocyte isolation

Peripheral blood mononuclear cells (PBMCs) were obtained from buffy coats by density gradient centrifugation using Ficoll-Paque PLUS (GE Healthcare Bioscience, Uppsala, Sweden).

NK-cells were isolated using RosetteSep™ Human NK-cell Enrichment Cocktail (Stem Cell Thecnology) by negative selection and centrifugation with RosetteSep™ DM-L density medium (Catalog #15705) to remove unwanted cells, according to the manufacturer’s instructions. After each separation, the enriched fraction was assessed by flow cytometry and the percentage of the CD56+CD3- cell population ranged from 87%.

Monocytes were obtained from PBMCs by immunomagnetic isolation using anti-CD14 microbeads and VarioMACS cell separator (Miltenyi Biotec), according to the manufacturer’s protocol. The purity of isolated population was always over 90%.

### MSC and T-cell coculture

Peripheral blood mononuclear cells were labelled with the intracellular fluorescent dye Carboxyfluorescein diacetate succinimidyl ester (CFSE, *CellTrace™ CFSE Cell Proliferation Kit;* Molecular Probe/Invitrogen, USA) at 5 mM, according to the manufacturer’s instructions. Before coculture, BM-MSCs were seeded in 24-well plates at a concentration of 10^5^ cells/well. Following overnight adherence, CKs were added during 24h. After a gently wash, 10^6^ stained PBMCs were added to wells with pre-stimulated or basal MSCs in the presence of T Cell TransAct™ (Miltenyi Biotech, Germany) by using a titer of 1:100 to induce the specific T-cell activation and expansion via CD3 and CD28, in RPMI 1640 medium supplemented with 10% fetal bovine serum and 1% penicillin/streptomycin (all from Gibco, Thermo Fisher). Stimulated and non-stimulated T-lymphocytes cultured without MSCs was carried out as control.

After 3 days of incubation, cells harvested from culture wells were analyzed for T cell proliferation by flow cytometry. Data were analyzed with ModFit LT™ (Verity Software House, USA).

Supernatants were collected and CK secretion was measured.

### MSC and NK-cell coculture

BM-MSCs were treated with the CK cocktail for 24h. After gently washed and trypsinization 10^5^, 10^4^ or 5x10^3^ MSCs per well were seeded in triplicate on 96-well plates in DMEM completed medium and allowed to adhere for 4 h.

Prior to coculture, NK-cells were stained with CFSE at 5 mM according to manufacturer’s instructions. Then, 10^5^ stained NK-cells were added to wells with CK-MSCs or basal MSCs in a final volume of 100µL to different MSC/NK-cells ratios 1:1, 1:10 and 1:20 in Stem Cell Growth medium (GMP SCGM, CellGenixt®), supplemented with 10% FBS and with 1% Penicillin/Streptomycin. NK-cells were stimulated with 20 ng/mL IL-15 (Miltenyi Biotec). After 5 days of coculture, non-adherent cells were collected and labelled with CD56-PE, 7AAD, CD3-PeCy7 and NKG2D-APC for 15 min in dark. Finally, proliferation and NKG2D expression were acquired in a FACSCanto II flow cytometry and the raw were analysed using the ModFit LT and Infinicyt softwares, respectively. Additionally, IL-12 (2ng/ml) and IL-18 (100ng/ml) were added to quantify CK release. After 48h, supernatants were collected, and the concentration of different CKs was measured.

### MSC and monocyte coculture

BM-MSCs were seeded in 6-well plates at a concentration of 5×10^5^ cells/well in 2 mL. Following overnight adherence, CK stimulus was added during 24h and then MSC cultures were gently washed. Monocytes were added at a ratio of 1:10 MSC/monocyte under the different conditions described below.

Monocytes were cultured in RPMI 1640 medium (Lonza) supplemented with 10% FBS (Gibco, Thermo Fisher), 100 U/mL penicillin, 100 µg/mL streptomycin, 2 mM L-glutamine and 1 mM pyruvate (all from Lonza), in the presence of GM-CSF (5 ng/mL) to induce M1 macrophages or with GM-CSF (20 ng/mL) and IL- 4 (20 ng/mL; all from Gibco. Thermo Fisher) to induce DC differentiation. After 3 days, additional 5 ng/mL GM-CSF was added to macrophage cultures and half of the medium was renewed in DC cultures. After 6 days of coculture, macrophage and DC phenotypes were analyzed by flow cytometry within the CD90^-^ population, using CD14 and CD163 antibodies for macrophages and CD1a and CD14 for DCs. The analysis was performed on a FACSCalibur flow cytometer (BD Biosciences) (Centre of Cytometry and Fluorescence Microscopy. Complutense University of Madrid) and analyzed using FCS Express V3 software.

Macrophages and DCs were stimulated overnight with LPS (Invitrogen, Life Technologies) at 10 ng/ml or 50 ng/ml, respectively. Supernatants were collected and DCs allostimulatory function was analyzed by culturing in mixed lymphocyte reaction (MLR) with CFSE-labelled T lymphocytes (1:10 DC/T cell ratio). After 5 days of coculture, T lymphocyte proliferation was analyzed in the CD3^+^ population using the CFSE dilution method by flow cytometry. Supernatants from different cocultures were harvested at different times and CK secretion was measured.

### Protein quantification

Proteins were measured in supernatants from BM-MSC or AT-MSC cultures, and BM-MSC cocultures with PBMCs, NK-cells or monocytes.

Supernatants from MSC cultures were collected 24 hours after cell pre-stimulation without removal of *priming* cytokines. These supernatants together with those collected from MSC-monocyte and MSC-PBMC cocultures were analyzed by flow cytometry using Human Essential Immune Response Panel (BioLegend, USA) according to the manufacturer’s instructions. The data obtained were analyzed using LEGENDplex Data Analysis Software Suite.

In MSC-NK cocultures, TNFα concentration was measured by Human Inflammatory Cytokine Cytometric Bead Array (BD Bioscience) according to the manufacturer’s instructions.

### Statistical analysis

The percentage of coefficient of variation and statistical analyzes were performed using Graph Pad Prism 9.0 software (Graph Pad Software, USA). The data were expressed as mean ± standard deviation (SD) or standard error of the mean (SEM), as indicated in the text and figures. Normal distribution was analyzed using Kolmogorov Smirnov test.

To compare two groups, statistical differences were determined by Wilcoxon test when the distribution was not normal or by paired *t-test* for normal distribution.

To compare more than two groups, parametric one-way ANOVA test was used for normal distribution, with subsequent Tukey’s *post hoc* analysis for multiple comparisons. When the distribution was not normal, the non-parametric Kruskal-Wallis’ test was performed with Dunn’s *post hoc* analysis for multiple comparisons. In this study, p-values < 0.05 were considered statistically significant: * p < 0.05; ** p < 0.01; *** p < 0.001; **** p < 0.0001.

## Results

### Proinflammatory priming of MSCs does not alter their differentiation potential, viability and phenotype.

We first analyzed whether stimulation with proinflammatory CKs modifies the osteogenic and adipogenic differentiation potential of MSCs. After the *in vitro* differentiation assays, they maintained the same capacity to differentiate into both lineages without differences between the experimental groups (**Supplementary Figures S1A-C**). Furthermore, the phenotype of MSCs was not significantly affected by preconditioning with proinflammatory CKs (data not shown). Moreover, MSCs and CK-MSCs showed similar viability after pre-stimulation, without altering the proliferative capacity of the cells (1.20x10^6^ ± 7.70x10^5^ cells and 1.11x10^6^ ± 5.35x10^5^ cells respectively) which resulted in a cell expansion about 2-fold (2.40 ± 1.54-fold for MSCs and 2.22 ± 1.07-fold for CK-MSCs) (**Supplementary Figures S1D-F**).

### Pre-stimulated MSCs display a different transcriptomic profile.

**Figure 1:**
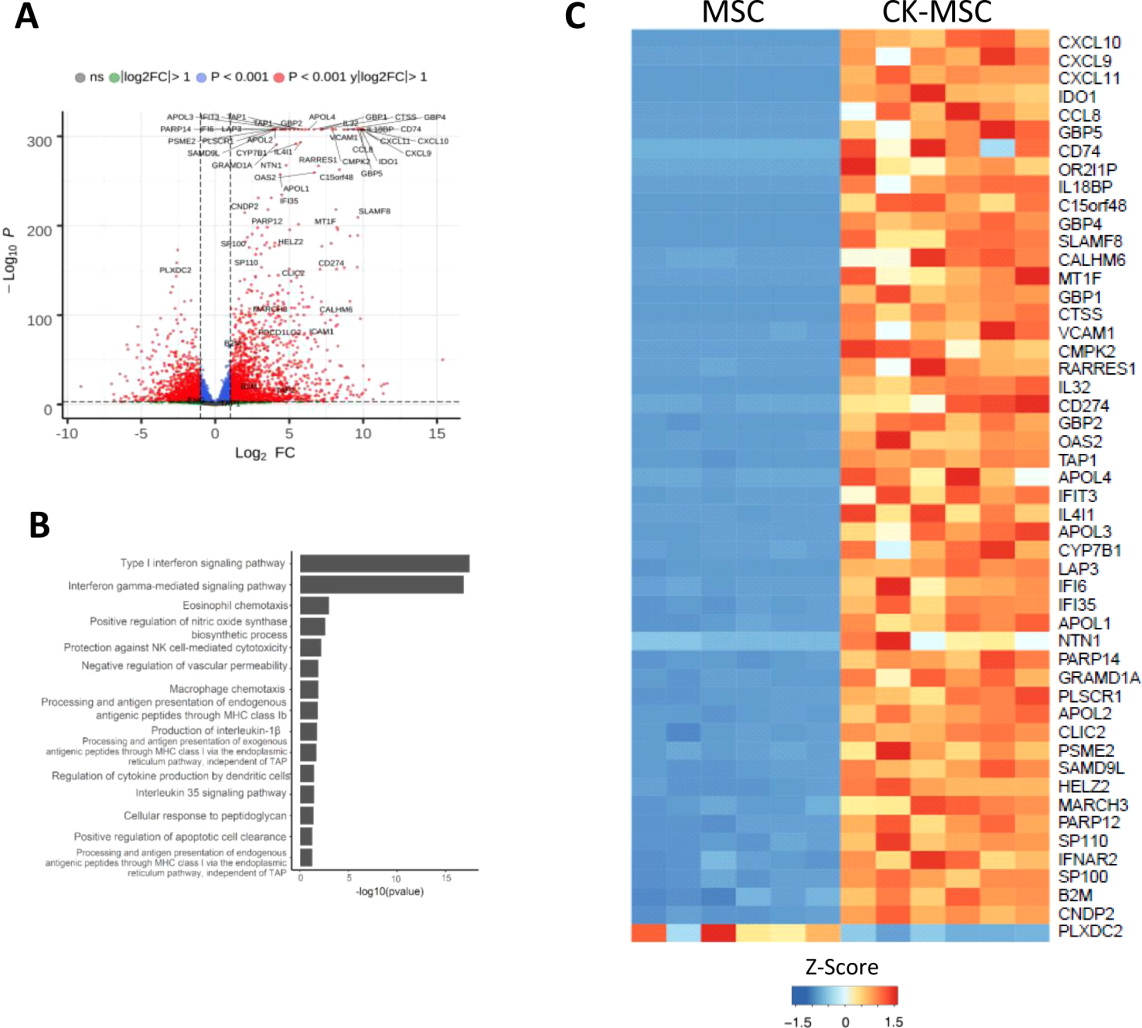
Transcriptome analysis of pre-stimulated and control MSCs by RNA-seq. **(A)** Differential gene expression in CK-MSCs as compared to MSCs in baseline conditions. Volcano plot show the distribution of the adjusted p values (-Log_10_ P) and the fold changes (Log_2_ FC). Significant changes are indicated in red (adjusted p-value <0.001 and a log2FC >|1|). **(B)** Go term plot of differentially expressed genes. **(C)** Heat map of the 50 most significant differentially expressed genes. (n=6).

To better understand the molecular changes induced in MSCs by the proinflammatory CKs stimulation, the transcriptome of stimulated and basal MSCs was compared by RNA-Seq. As shown in **Figure 1A**, 1,310 genes had an adjusted p-value <0.001 and a log2FC >|1|. Among these 1,310 genes, 923 showed increased expression, while 387 downregulated their expression in CK-MSCs (**Figure 1A**). Functional categories of the differentially expressed genes were obtained using Gene Ontology (**Figure 1B**). Notably, after organizing the categories by adjusted *p-value*, the differentially expressed genes were mostly related to type I interferon and IFNγ signaling pathways. In addition, genes involved in macrophage and eosinophil chemotaxis and the regulation of CKs released by DCs and NK-cells were also overrepresented. The top 50 differentially expressed genes are shown in **Figure 1C**. They include interferon-induced inflammatory chemokines such as *CXCL10, CXCL9, CXCL11* and *CCL8*, and interferon-induced proteins such as *IFI10*, *IFI35* and *IFIT3*. Also, immunomodulatory factors such as *IDO1* or *CD274* (PD-L1) and adhesion molecules (i.e., *VCAM1*). In general, the differentially expressed genes belong mainly to functional categories related to inflammation and the immune system. These RNA-seq data have been deposited in the GEO NCBI database with the accession https://dataview.ncbi.nlm.nih.gov/object/PRJNA1129604?reviewer=d8leo6cd7l3c13uecl8ifbflc6.

### Priming with proinflammatory CKs reduces the heterogeneity in the expression of immunomodulatory genes between MSCs derived from different donors

**Figure 2:**
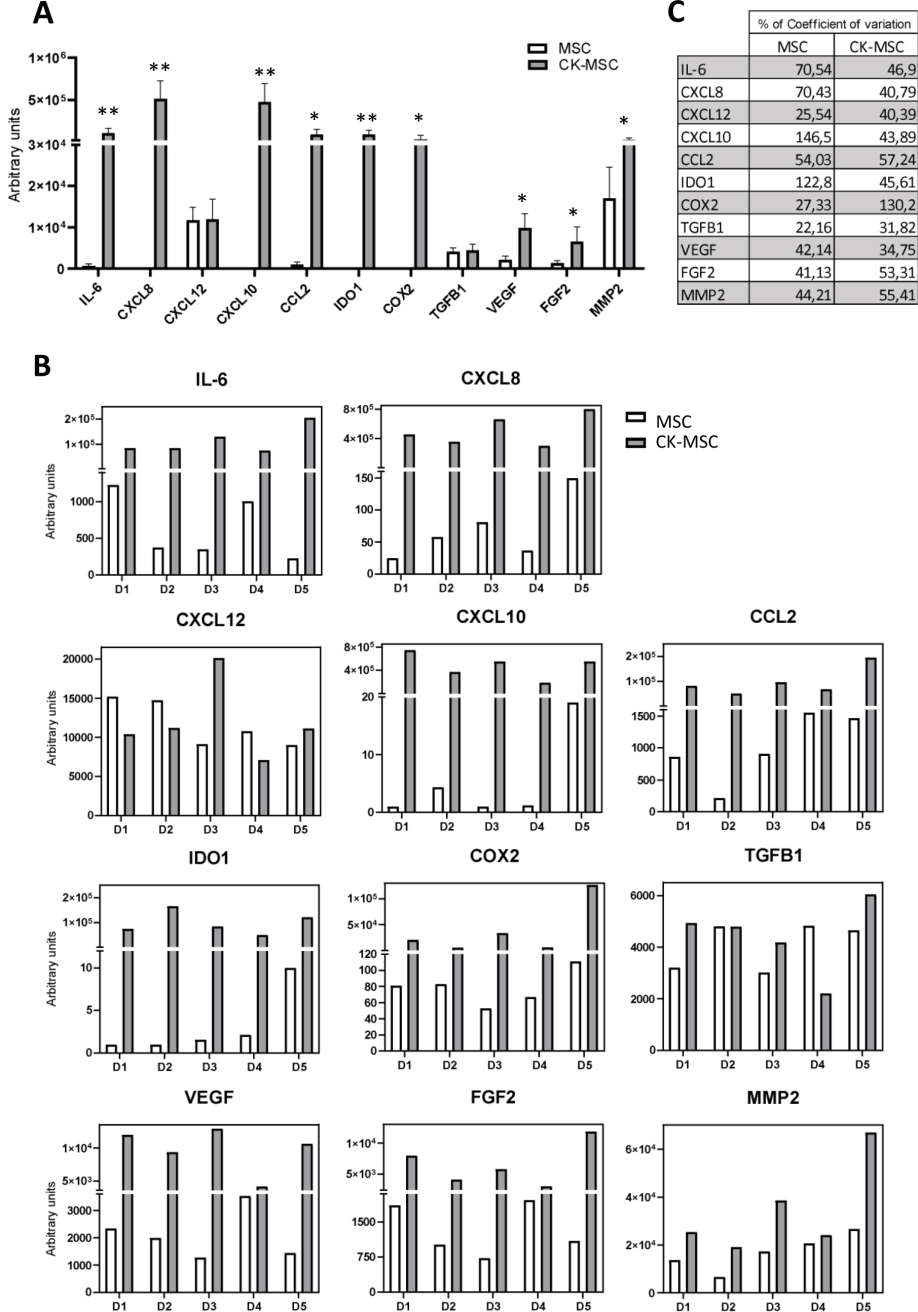
Priming with proinflammatory CKs reduces the differences in MSC gene profile between different donors. **(A, B)** mRNA expression for different immunomodulatory factors on MSCs and CK-MSCs by qRT-PCR. **(A)** Data represent mean ± SD of 5 independent donors. *p<0.05; **p<0.01 by paired t-test. **(B)** Mean of mRNA expression in each individual donor (D1-D5). **(C)** Percentage of coefficient of variation of gene expression between 5 independent donors of MSCs.

Real-time PCR confirmed the upregulated expression of genes involved in the immunoregulatory capacity of MSCs after priming. As shown in **Figure 2A**, *IL-6, IL-8, CXCL10, CCL2, IDO-1, COX2, VEGFA, FGF2* and *MMP2* expression was increased. However, the expression of *CXCL12* and *TGFβ1* did not change significantly.

Most importantly, our results showed that proinflammatory priming reduced the differences in MSCs gene expression profile between different donors (**Figure 2B**). Moreover, as shown in **Figure 2C**, the percentage of coefficient of variation of gene expression was markedly reduced in CK-MSCs compared to baseline MSCs for the most analyzed genes. For instance, the expression of *CXCL10* and *IDO1*, genes that were significantly upregulated in the RNA-Seq analysis, showed an important decreased in their coefficient of variation upon CK stimulation (**Figures 2B, C**).

### Pre-stimulated MSCs show an increased immunomodulatory capacity mediated by secreted factors and cell to cell contact

**Figure 3:**
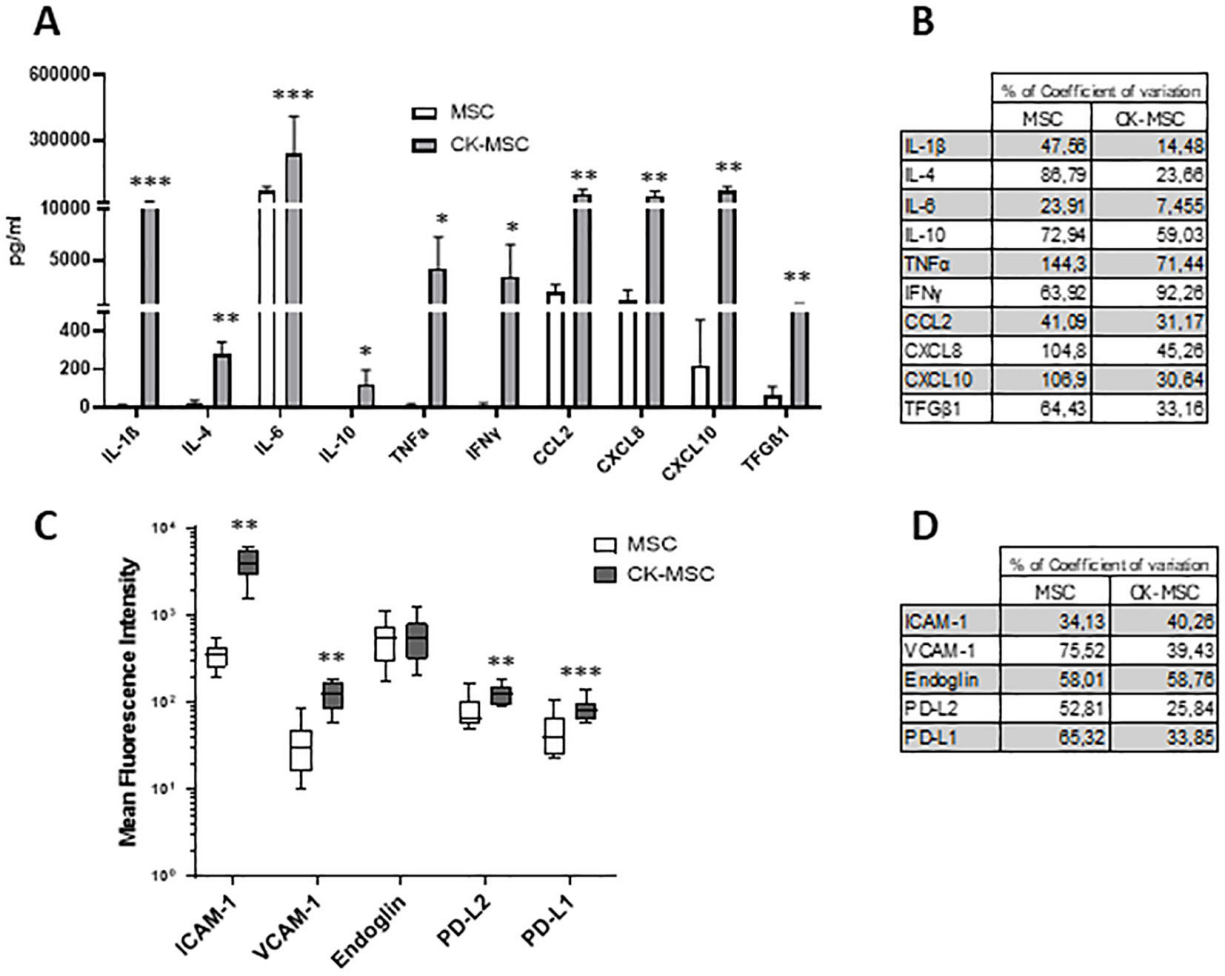
**(A)** Pre-stimulated MSCs significantly increased secretion of CKs and chemokines compared to control MSCs, measured in culture supernatants after 24h of pre-stimulation without removal of priming cytokines. Bars represent mean ± SD of 5 independent experiments. **(B)** Percentage of coefficient of variation of protein secretion between 5 independent donors of MSC. **(C)** Expression of immunoregulatory and adhesion molecules in control and pre-stimulated MSCs analyzed by flow cytometry (n=6). **(D)** Percentage of coefficient of variation of immunoregulatory and adhesion molecules between 6 independent donors of MSCs *p<0.05; **p<0.01; ***p<0.005 by paired t test.

Flow cytometry analysis of CKs and chemokines present in the MSCs culture supernatants showed that CK-MSCs significantly increased the secretion of IL-1β, IL-4, IL-6, IL-10, TNFα, IFNγ, CCL2, CXCL8, CXCL10 and TFGβ1 compared to untreated, basal MSCs (**Figure 3A**). As observed in the gene expression profile of MSCs (**Figures 2B, C**), proinflammatory priming led to a more homogeneous pattern, with a lower percentage of coefficient of variation observed between samples from different donors with respect to the secretion of relevant immunomodulatory proteins (**Figure 3B**). Similar results were observed when AT-MSCs were activated with proinflammatory CKs (**Supplementary Figure S2A, B**).

In the same way, flow cytometry analysis revealed positive upregulation of the membrane molecules ICAM-1, VCAM-1, PDL2 and PDL-1 after MSC priming, and confirmed that the use of CKs to activate MSCs reduces inter-donor variability (**Figure 3C, D**).

### Effects on pre-stimulated-MSC are maintained over time and in a proinflammatory setting

**Figure 4:**
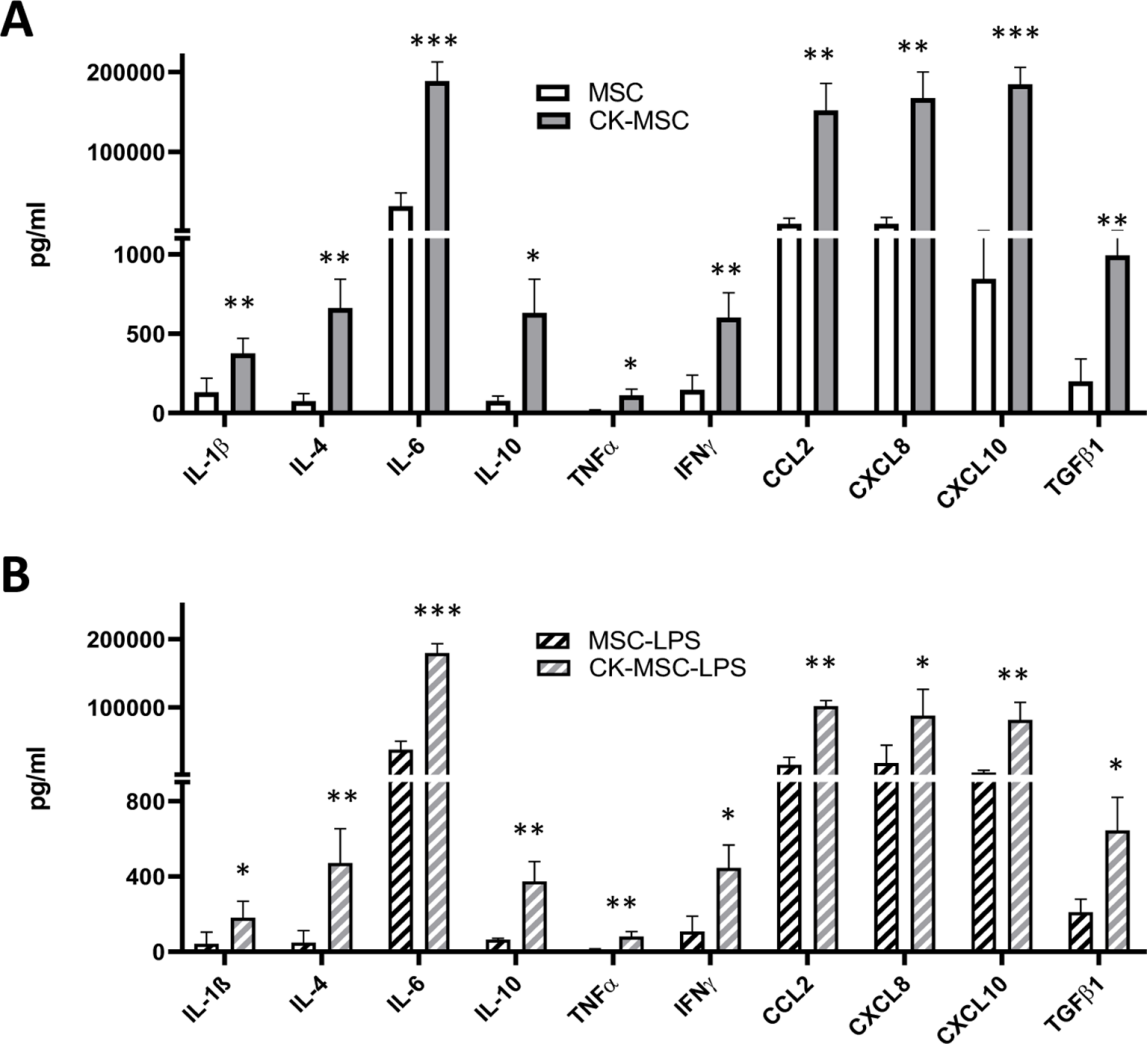
Effects on pre-stimulated-MSCs are maintained over time and in a pro-inflammatory environment. **(A)** Protein secretion by MSCs 5 days after priming. **(B)** Protein secretion by MSCs after 24h LPS stimulation, 5 days after priming (n=4). *p<0.05; **p<0.01; ***p<0.005 by paired t test.

Then, we evaluated whether the response of MSCs to proinflammatory CKs was transient or could be maintained over time and after re-stimulation, mimicking the natural history of most inflammatory diseases. As shown in **Figure 4A**, five days after priming and gentle washing of the cultures to remove the CK stimulus, CK-MSCs maintained a significantly higher level of immunomodulatory protein secretion compared to non-pre-stimulated MSCs with values similar to those found at 24h (**Figure 3A**), except for the CKs IL-1β, TNFα and IFNγ, which were present in the proinflammatory priming cocktail.

In addition, when we simulated an inflammatory microenvironment by adding LPS to the MSC culture during 24h, five days after priming, we observed that the effect on CK-MSCs was maintained even after a secondary inflammatory stimulus (**Figure 4B**).

### Effect of pre-stimulated MSCs on T cell proliferation and function

**Figure 5:**
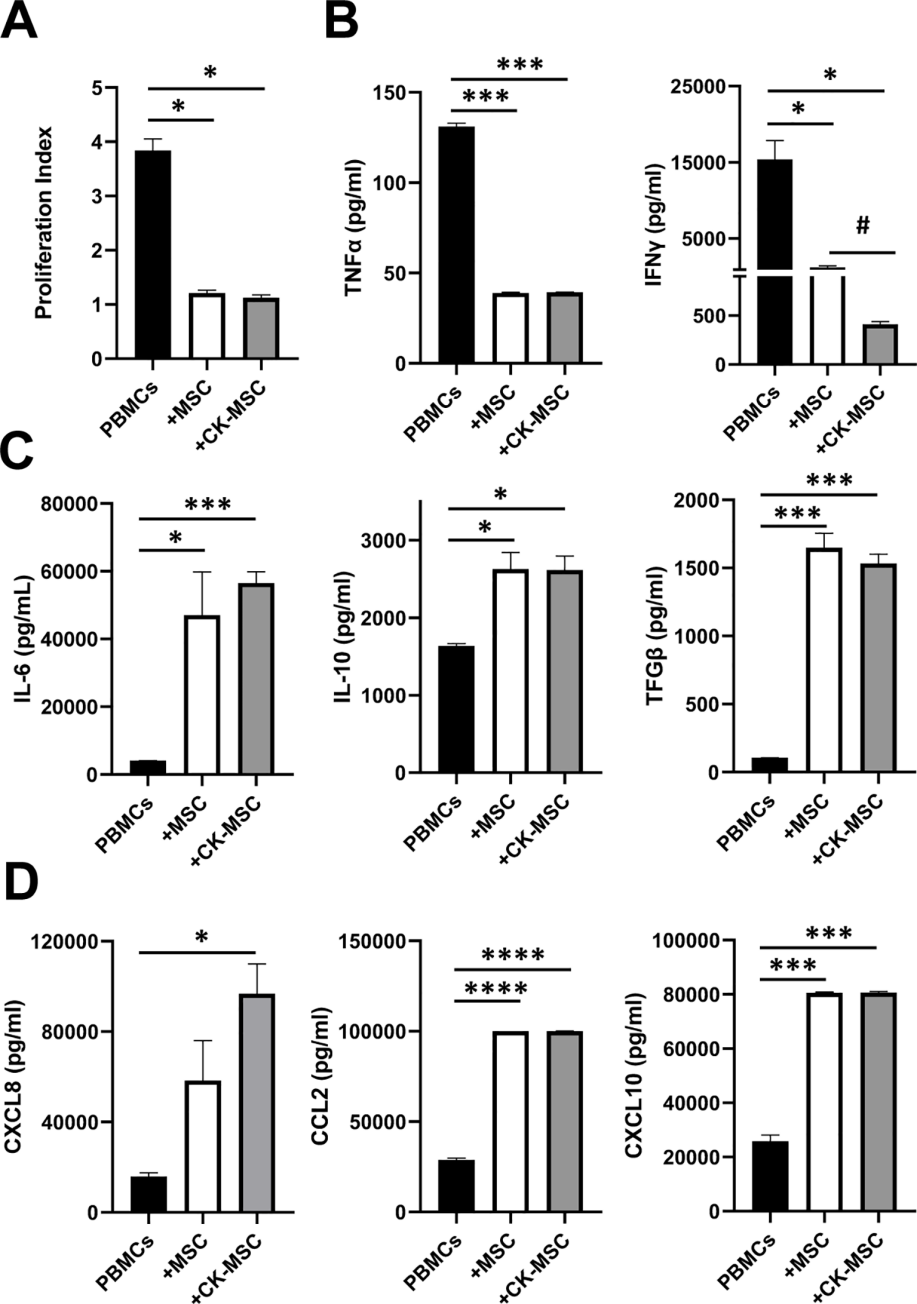
Effect of pre-stimulated MSCs on T-cell proliferation and function. PBMCs were labelled with CFSE, incubated with anti-CD3 and anti-CD28 and cultured in absence or presence of MSCs or CK-MSCs for 3 days. **(A)** T-cell proliferation was analyzed by flow cytometry. **(B-D)** CKs and chemokines levels measured in supernatants of cocultures. Data represents the mean ± SEM of three to four independent experiments. *p<0.05; ***p<0.005; ****p<0.0001 significances relative to PBMCs; ^#^p<0.05 significance relative to MSC by paired t test.

We next analyzed the capacity of pre-stimulated MSCs to inhibit T cell proliferation compared to baseline MSCs. As shown in **Figure 5A**, T cells proliferation was significantly reduced by both types of MSCs, with no significant differences between them.

Also, we analyzed in the supernatants of the cocultures, the levels of different CKs and chemokines important for T cell function. As shown in **Figure 5B**, both types of MSCs markedly reduced the production of inflammatory CKs like TNFα and IFNγ by T cells compared to the levels detected in T cell cultures. However, a significant reduction in the levels of IFNγ (but not of TNFα) in cocultures with CK-MSCs compared to control MSCs was observed. When levels of CKs and soluble factors such as IL-6, IL-10, TFGβ1, CCL2 and CXCL10 (involved in the MSC-mediated immunosuppression) were analyzed, all of them were significantly increased by both experimental groups, without differences between them. However, the secretion of CXCL8 was significantly increased in CK-MSCs (**Figures 5C, D**).

Although CK activation of MSCs did not improve the ability of MSCs to control T cell proliferation, our results showed that CK-MSCs inhibited T cell proliferation at least as efficiently as MSCs, reducing the levels of proinflammatory CKs and increasing the secretion of immunomodulatory molecules.

### Pre-stimulated-MSC showed an increased immunomodulatory capacity on NK-cells function

**Figure 6:**
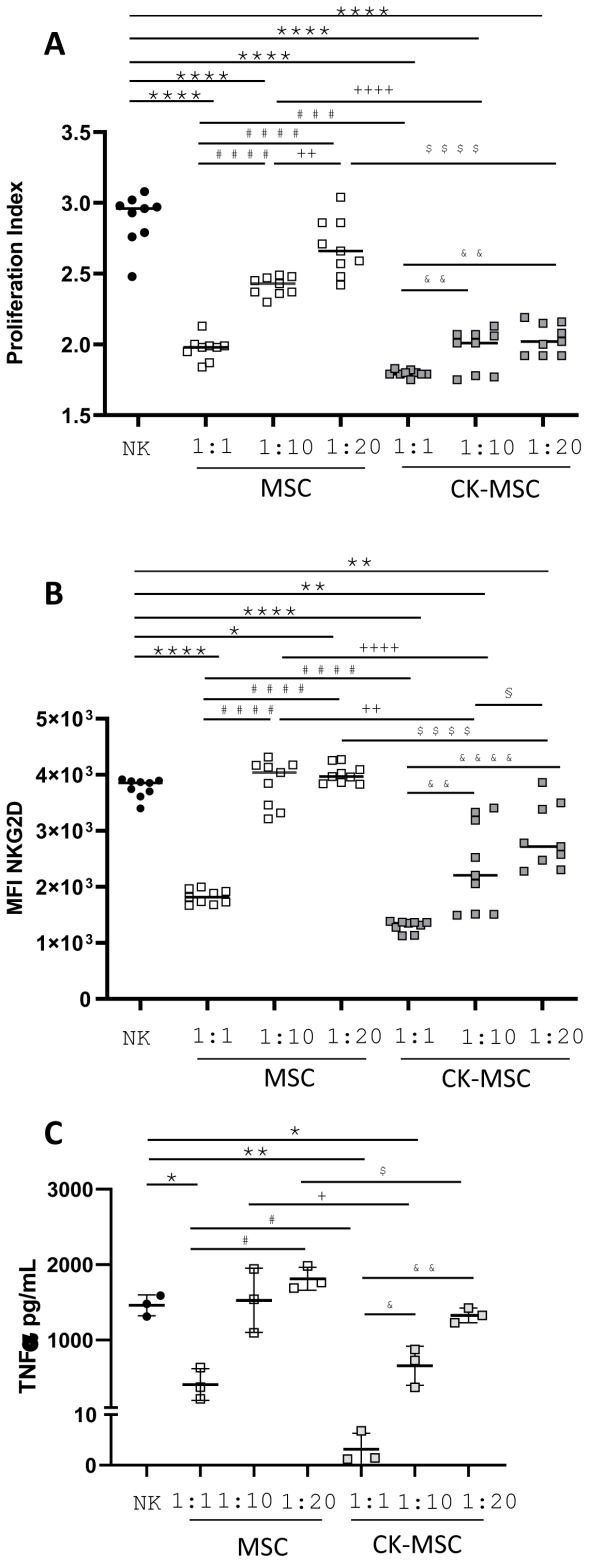
Effect of pre-stimulated MSCs on NK-cell function. NK-cells were stimulated with IL-15 and culture in the absence or presence of different ratios of MSCs or CK-MSCs for 5 days. **(A)** Proliferation indexes were measured by CFSE dilution method by flow cytometry. **(B)** Mean fluorescence Intensity of NKG2D expression on NK-cells. **(C)** TNFα secretion was measured in the supernatants of MSC: NK-cell cocultures (n= 3-9) *p<0.05; **p<0.01; ****p<0.0001 significances relative to NK-cells; ^#^ significance relative to 1:1 MSC:NK ^#^p<0.05; ^###^p<0.005; ^####^p<0.0001; ^+^ significance relative to 1:10 MSC:NK ^+^p<0.05; ^++^p<0.01; ^++++^p<0.0001; ^$^ significance relative to 1:20 MSC:NK ^$^p<0.05; ^$$$$^p<0.0001; ^&^ significance relative to 1:1 CK-MSC:NK ^&^p<0.05; ^&&^p<0.01; ^&&&&^p<0.0001; ^§^ significance relative to 1:10 CK-MSC:NK ^§^p<0.05 by paired t test.

As already mentioned in the methodology section, MSC: NK-cell cocultures were performed at different concentrations. As shown in **Figure 6A**, basal MSCs at low concentrations (1:20) did not significantly decrease NK proliferation. However, CK-MSCs at the same concentration reduced NK proliferation to levels comparable to the highest concentration (1:1) of MSCs cultured without stimulation. Moreover, our results showed that CK-MSCs reduced donor variability on the decrease of NK-cell proliferation (see ratio 1:20 and 1:1). Similarly, our results showed that lower ratio of CK-MSCs than MSCs was sufficient to significantly decrease the level of expression of the NKcells activating receptor NKG2D (1:20) (**Figure 6B**). Furthermore, the reduction of TNFα levels was more pronounced in culture with CK-MSCs than MSCs, even at lower ratios (1:10) (**Figure 6C**).

### Effects of MSC priming on myeloid cell differentiation and function

**Figure 7:**
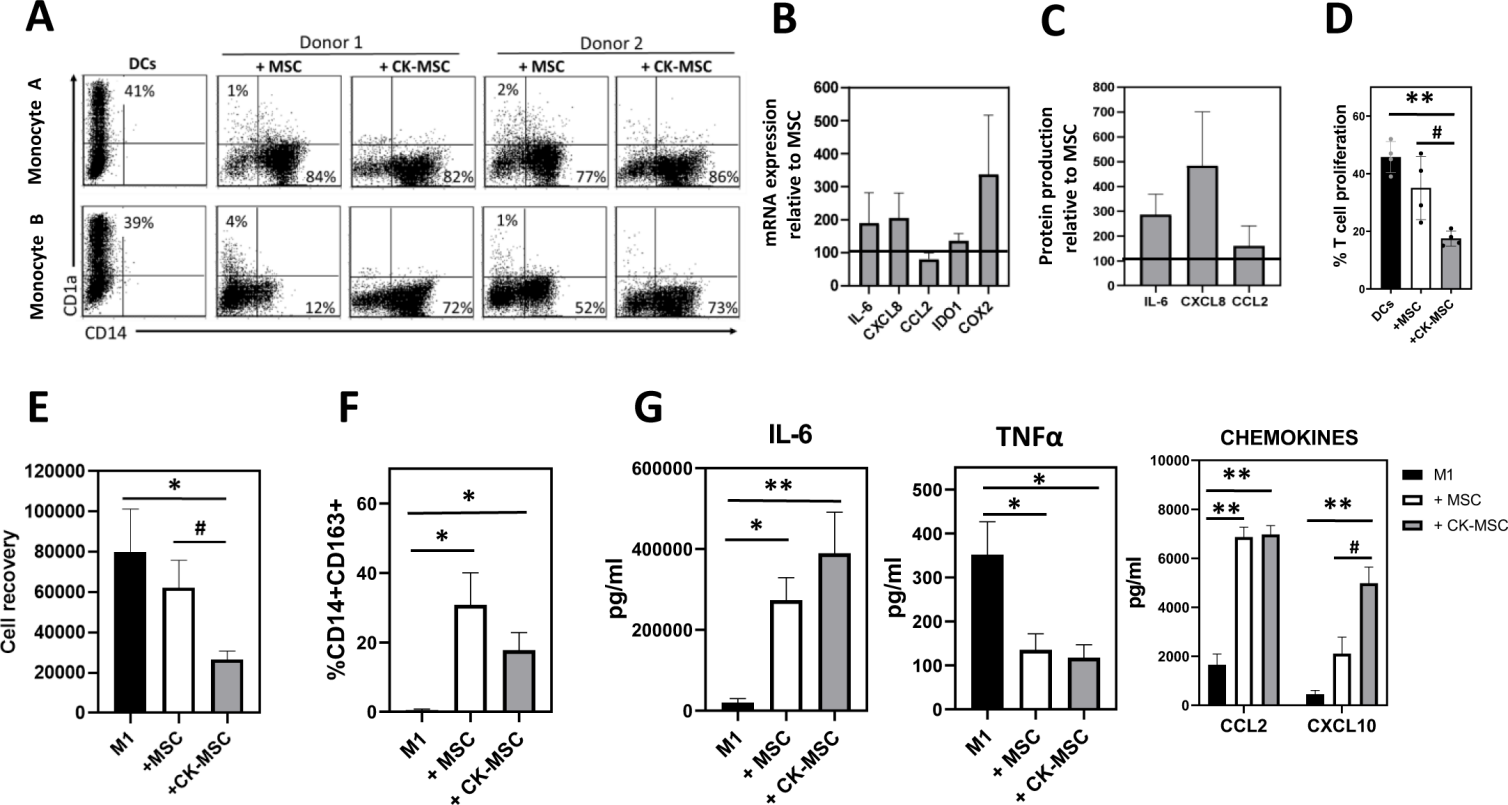
Effects of MSC priming on myeloid cells differentiation and function. CD14+ monocytes were cultured for 6 days with GM-CSF and IL- 4 to induce DC differentiation **(A-D)** or with GM-CSF to induce M1 macrophages **(E-G)** in the absence or presence of MSC or CK-MSC. **(A)** CD1a and CD14 expression on DCs analyzed by flow cytometry. The percentage of CD1a+CD14- (DCs) and CD14+ CD1a- (monocytes) are shown in each plot (within the CD90- population). We show representative data from two different MSC donors cocultured with two different samples of monocytes. **(B)** mRNA levels and **(C)** protein secretion of different immunomodulatory factors by DCs cocultured with CK-MSC related to those cocultured with MSC (referred as 100). **(D)** DCs stimulated with LPS were cultured in MLR assay with CFSE-labeled T cells. After 5 days, the percentage of proliferating T cells was calculated by CFSE dilution method. **(E)** Number of macrophages recovered, **(F)** percentage of M2-like CD14+CD163+CD90- cells and **(G)** CKs and chemokines production by macrophages were measured after 6days of culture in the absence (M1) or presence of MSC or CK-MSC. Mean ± SEM of four independent experiments is shown. *p<0.05; **p<0.01 significances relative to DCs or M1; ^#^p<0.05 significance relative to MSC by paired t test.

To investigate the effect of CK priming on MSC capacity to modulate monocyte differentiation, we carried out cocultures of MSCs, pre-stimulated or not, with monocytes driven to differentiate into dendritic cells (DC) or macrophages (**Figure 7**).

Our results showed that CK-MSCs inhibited DC differentiation from monocytes at least as efficiently as MSCs under basal conditions. However, pre-stimulated MSCs were able to significantly reduce the differences in the inhibition of DC differentiation due to both, MSC-donor and MSC-monocyte interactions (**Figure 7A**). Additionally, DCs differentiated in the presence of pre-stimulated MSCs produced higher levels of IL6, IDO and COX2, which are relevant for their tolerogenic activity (**Figures 7B, C**). Otherwise, CXCL8 and CCL2 were also increased in these cocultures (**Figure 7C**). Next, we analyzed the functional capacity of DCs generated under these conditions as stimulators of allogeneic T cells. As shown in **Figure 7D**, CK-MSCs reduced the allostimulatory capacity of DCs more efficiently than MSC under baseline conditions. Therefore, DCs generated in the presence of CK-MSCs produce higher levels of tolerogenic factors and show a reduced ability to induce T cell proliferation.

Then, we examined whether proinflammatory priming could modify the effects of MSCs on the repolarization of monocytes towards M2-like macrophages. First, we observed that M1-macrophages recovery was significantly decreased in cocultures with CK-MSCs (**Figure 7E**). In addition, after 6 days of differentiation, the percentage of M2-like CD14+CD163+ cells was increased in cocultures with CK-MSC, similar to MSC in control conditions (**Figure 7F**). Finally, we analyzed the CK and chemokine production of these cells after LPS activation. As shown in **Figure 7G**, M2-like cells generated in the presence of pre-stimulated MSC secreted reduced levels of the proinflammatory CK TNFα and increased levels of immunomodulatory factors such as IL-6, CCL2 similar to MSCs in baseline conditions. However, the increased amount of CXCL10 was significantly higher in the cocultures with CK-MSCs than with non-pre-stimulated MSCs. Thus, pre-stimulated MSCs would facilitate the recruitment of monocytes which would then acquire immunosuppressive properties.

## Discussion

*In vivo* priming by inflammatory CKs is essential for MSC-mediated immunomodulation (13, 27). In this study, we have multiparametrically assessed analyze the changes in the immunomodulatory profile of CK- MSCs compared to baseline MSC and their effects on their *in vitro* interactions with key elements of the immune system. In addition, we have tested the effects after a second inflammatory challenge, which is important, since the clinical evolution of many of the inflammatory and immune diseases that have been treated with MSCs is in flares or acute episodes of inflammation/autoimmunity.

Differentiation potential of MSCs is a key feature for their therapeutic application in osteoarticular diseases. Our results show that priming does not modify osteogenic and adipogenic differentiation potential of MSCs, although there is some controversy regarding the effect of proinflammatory CK priming on the osteogenic and adipogenic potential of MSCs, since both enhancing and decreasing effects have been reported (28) likely due to the different combination of inflammatory CKs used in MSCs preconditioning. Some authors have showed that IL-1β and TNFα suppressed osteogenic (29) and adipogenic differentiation (30) of BM-MSCs. However other studies have shown that TNFα stimulation of BM and AT-MSCs upregulates osteogenic factors (31). Furthermore, Duijvestein et al. reported that treatment of MSCs with IFNγ does not alter the differentiation potential of MSCs into osteoblasts, adipocytes and chondroblasts (32). Likewise, in our cultures, CK priming did not modify MSCs viability or phenotype therefore suggesting that generated CK-MSCs retain the basic self-renewal and stemness properties of MSCs.

Interestingly, our results point out that the response to priming with inflammatory CKs is similar in AT-MSCs and BM-MSCs, suggesting that the response to priming is independent of the cell source. Moreover, our results confirm that CK priming extensively modifies MSC gene expression, showing increased expression of immune and inflammation-related genes, especially those involved in IFN signaling pathways. In agreement with our results, RNA-Seq analysis by Herger et al. indicates that proinflammatory priming of MSCs leads to up-regulation of genes involved in the immune system response and in the defense response against viruses (33). In addition, mRNA and protein assessment of our cultures indicate that most of the immunoregulatory factors analyzed are significantly upregulated after CK priming such as IL-6, CXCL8, CXCL10, CCL2. Furthermore, similar to that was described by Gómez-Ferrer et al, CK-MSCs show increased expression of key immunosuppressive factors such as IDO1, COX2, PDL1 and PDL2 in CK-MSCs (34). The upregulation of pro- and anti-inflammatory cytokines after MSCs pre-stimulation is consistent with those observed in various studies which have shown that IFNγ, TNFα and/or IL1β regulates NF-κB and MAPK signaling pathways (35–40), involved in the expression of cytokines such as IL-6, IL-10, CCL2, CXCL8, CXCL10 or COX2 (40–42). As different studies have previously shown, the increase in both proinflammatory and anti-inflammatory cytokines could play an important role in immunomodulation, due to the dynamics and interaction network between both types of cytokines in the regulation of the immune system by cellular, immunological and biochemical mediators in different pathologies and infectious processes (43–45).

Our study also shows for the first time that proinflammatory priming reduces the heterogeneity in gene and protein expression between MSCs from different donors and exhibit a more consistent immunomodulatory activity. Recently, great efforts have been made to reduce the variability of MSC, homogenizing MSC isolation and expansion protocols, standardizing their cryopreservation, routes of administration, etc. (25, 46). Therefore, one of our main conclusions is that priming with proinflammatory CKs could represent a good strategy to standardize, homogenize and therefore improve clinical outcomes after MSCs therapy.

Regarding the immunomodulatory function of MSCs, it is well established that MSCs exert an inhibitory effect on the proliferation, differentiation, and function of different types of immune cells from both innate and adaptive immunity (9, 47, 48). In addition, several studies have demonstrated that the effect of proinflammatory CK priming improves the immunosuppressive function of MSCs (13, 24, 28, 49). However, the immunomodulatory capacity of MSCs after priming with proinflammatory CKs, on both innate and adaptive immune cell differentiation, proliferation and function comprehensively assessed within the same work has not been performed to date and is another key feature of our current study.

The immunosuppressive role of MSCs on T-cell proliferation and function is well established (50, 51). MSCs suppress T-cell proliferation (52). In addition, preconditioning of MSCs with different strategies enhances their ability to inhibit T-cell function, mainly through upregulation of IDO1, iNOS and other immunomodulatory factors (13, 24). However, in agreement with our results, Chinnadurai et al. described that both resting and IFN-γ–licensed MSCs show an equivalent ability to block T-cell proliferation *in vitro*, with non-preconditioned AT-MSCs able to achieve maximum inhibition (53). Although in this work we did not observe an increase in the immunomodulatory capacity of CK-MSCs against T cells, probably due to the high efficiency of non-pre-stimulated MSCs in almost completely controls T-cell proliferation, there is a tendency for CK-MSCs to induce a reduction in pro-inflammatory CKs released by T-cells, with a significant decrease in IFNγ, but slightly increased levels of IL-6, IL-10 and CXCL8.This could correlate with previous studies in which pre-stimulation of MSCs with IFNγ was shown to reduce the pro-inflammatory cytokines secretion and increase the secretion of anti-inflammatory cytokines, more effectively reducing the proportion of Th1 cells and increasing the proportion of Th2 and Treg cells (54, 55).

NK-cell proliferation and CK production are differentially regulated and modulated by MSCs depending on NK-cell-activating CKs. Furthermore, the presence of MSCs significantly inhibited NK-cell proliferation, but to different levels, and this inhibition was dependent on the NK/MSC ratio (56). Few studies have analyzed the effect of proinflammatory priming of MSCs on their NK-cell immunoregulatory function. Noone et al. showed that IFN-γ-preconditioned MSCs suppressed NK activation more efficiently than non-preconditioned MSCs. In addition, some studies have shown that IFN-γ priming protects MSCs from lysis by NK-cells as a result of the upregulation of HLA class I molecules on the surface of MSCs (57). Our results show that CK-MSCs are more efficient at inhibiting NK-cells proliferation and function than MSCs under baseline conditions, with pronounced effects even at high NK/MSC ratios. Similar results have been recently reported by Li et al. using umbilical cord MSCs exposed to hypoxia and inflammatory factors (25).

Continuing the effect of MSCs on innate immune cells, MSCs can inhibit the differentiation of DCs from both hematopoietic stem cells and monocytes (58, 59). In addition, MSCs impair DCs maturation and switch their CK profile from pro-inflammatory to immunoregulatory, reducing their ability to activate T cells. However, the effect of preconditioning on the role of MSCs in dendritic cell differentiation and function has not been fully elucidated. In this regard, our results show that priming with proinflammatory CK increases the ability of MSCs to inhibit DC allostimulatory function resulting in reduced activation of the adaptive immune response. Moreover, MSCs priming significantly reduces the differences in inhibition of DC differentiation by both MSCs-donor and MSCs-monocyte interaction.

There is increasing evidence that macrophages are critical for maintaining tissue homeostasis, particularly during tissue repair after inflammation. Moreover, many immune disorders, including inflammatory bowel disease (60), systemic lupus erythematosus (61) or wound healing (62), among others, have been reported as macrophage-mediated disorders. Therefore, the study of the role of MSCs in macrophage differentiation and function during tissue homeostasis and damage repair has received increasing attention in recent years (63, 64). Many studies have reported that MSCs can induce clinical responses in different diseases by polarizing macrophages from proinflammatory M1 cells to anti-inflammatory M2 cells (65–67). Additionally, some authors have reported that the pretreatment of MSCs with different combinations of proinflammatory CKs enhances their ability to induce M2 polarization, accelerating wound healing (68, 69) or improving experimental Crohn’s disease, among others (34). In this regard, our results show that in the presence of CK-MSCs, the number of M1 macrophages is significantly reduced compared to MSCs under baseline conditions. In addition, cells generated under these conditions exhibit M2 features, secreting lower levels of TNFα and higher levels of immunomodulatory molecules. Finally, CXCL8 and CCL2 are also increased in these cocultures, which could be responsible for the recruitment of more leukocytes to the area, including monocytes that can differentiate into new DCs and M2-like macrophages. Accordingly, our results suggest that the main effect of proinflammatory CK preconditioning of MSCs is on innate immune cells, which have been also described by several authors as the main mediators of the immunomodulatory effect of MSCs in the treatment of different diseases (70, 71).

Finally, in contrast to the recent publication by Herger and coworkers showing that the transcriptomic changes of pre-stimulated MSCs fade rapidly (33), our results show that the effect of CK priming of MSCs on the secretion of immunomodulatory factors is maintained over a period of time and, more importantly, after a subsequent inflammatory boost. In this sense, we and others have previously reported that changes in the immunomodulatory profile of MSCs pre-stimulated with TLR ligands are also maintained over time and after a second inflammatory stimulus (72, 73). Therefore, our results suggest that MSC response to CK priming is not transient but sustained in an inflammatory microenvironment, which is relevant for their use in advanced therapies for the treatment of inflammation-mediated diseases, that tend to course with flares.

## Conclusion

Our study confirms that *in vitro* proinflammatory priming of MSCs enhances their immunomodulatory capacity in both innate and adaptive immune cells without compromising their phenotype, viability and differentiation potential. Most notably, our results show that the functional variability between MSCs from different donors and sources is reduced after priming with proinflammatory CKs. Furthermore, the enhanced immunomodulatory secretion capacity of pre-stimulated MSCs persists over time and after receiving a second inflammatory stimulus.

## Data Availability

The datasets presented in this study can be found in online repositories. The names of the repository/repositories and accession number(s) can be found below: PRJNA1129604 (SRA).

## References

[1] Zhuang W-Z Lin Y-H Su L-J Wu M-S Jeng H-Y Chang H-C. Mesenchymal stem/stromal cell-based therapy: mechanism, systemic safety and biodistribution for precision clinical applications. J Biomed Sci. (2021) 28:28. doi: 10.1186/s12929-021-00725-7 33849537 PMC8043779

[2] Jimenez-Puerta GJ Marchal JA López-Ruiz E Gálvez-Martín P. Role of mesenchymal stromal cells as therapeutic agents: potential mechanisms of action and implications in their clinical use. J Clin Med. (2020) 9:445. doi: 10.3390/jcm9020445 32041213 PMC7074225

[3] García-Bernal D García-Arranz M Yáñez RM Hervás-Salcedo R Cortés A Fernández-García M. The current status of mesenchymal stromal cells: controversies, unresolved issues and some promising solutions to improve their therapeutic efficacy. Front Cell Dev Biol. (2021) 9:650664. doi: 10.3389/fcell.2021.650664 33796536 PMC8007911

[4] Han Y Yang J Fang J Zhou Y Candi E Wang J. The secretion profile of mesenchymal stem cells and potential applications in treating human diseases. Signal Transduct Target Ther. (2022) 7:92. doi: 10.1038/s41392-022-00932-0 35314676 PMC8935608

[5] DiMarino AM Caplan AI Bonfield TL. Mesenchymal stem cells in tissue repair. Front Immunol. (2013) 4. doi: 10.3389/fimmu.2013.00201 PMC3761350 24027567

[6] Sagaradze GD Basalova NA Efimenko AY Tkachuk VA. Mesenchymal stromal cells as critical contributors to tissue regeneration. Front Cell Dev Biol. (2020) 8:576176. doi: 10.3389/fcell.2020.576176 33102483 PMC7546871

[7] Klinker MW Wei CH. Mesenchymal stem cells in the treatment of inflammatory and autoimmune diseases in experimental animal models. World J Stem Cells. (2015) 7:556–67. doi: 10.4252/wjsc.v7.i3.556 PMC4404391 25914763

[8] Fernández-Garza LE Barrera-Barrera SA Barrera-Saldaña HA. Mesenchymal stem cell therapies approved by regulatory agencies around the world. Pharm (Basel). (2023) 16:1334. doi: 10.3390/ph16091334 PMC10536665 37765141

[9] Song N Scholtemeijer M Shah K. Mesenchymal stem cell immunomodulation: mechanisms and therapeutic potential. Trends Pharmacol Sci. (2020) 41:653–64. doi: 10.1016/j.tips.2020.06.009 PMC7751844 32709406

[10] Gao Q Jia F Li X Kong Y Tian Z Bi L. Biophysical cues to improve the immunomodulatory capacity of mesenchymal stem cells: The progress and mechanisms. Biomed Pharmacother. (2023) 162:114655. doi: 10.1016/j.biopha.2023.114655 37031489

[11] Roemeling-van-Rhijn M Mensah FK Korevaar SS Leijs MJ van Osch GJ Ijzermans JN. Effects of hypoxia on the immunomodulatory properties of adipose tissue-derived mesenchymal stem cells. Front Immunol. (2013) 4:203. doi: 10.3389/fimmu.2013.00203 23882269 PMC3714546

[12] Ren G Zhang L Zhao X Xu G Zhang Y Roberts AI. Mesenchymal stem cell-mediated immunosuppression occurs via concerted action of chemokines and nitric oxide. Cell Stem Cell. (2008) 2:141–50. doi: 10.1016/j.stem.2007.11.014 18371435

[13] Noronha NC Mizukami A Caliári-Oliveira C Cominal JG Rocha JLM Covas DT. Priming approaches to improve the efficacy of mesenchymal stromal cell-based therapies. Stem Cell Res Ther. (2019) 10:131. doi: 10.1186/s13287-019-1224-y 31046833 PMC6498654

[14] Waterman RS Tomchuck SL Henkle SL Betancourt AM. A new mesenchymal stem cell (MSC) paradigm: polarization into a pro-inflammatory MSC1 or an Immunosuppressive MSC2 phenotype. PloS One. (2010) 5:e10088. doi: 10.1371/journal.pone.0010088 20436665 PMC2859930

[15] Kebriaei P Hayes J Daly A Uberti J Marks DI Soiffer R. A phase 3 randomized study of remestemcel-L versus placebo added to second-line therapy in patients with steroid-refractory acute graft-versus-host disease. Biol Blood Marrow Transplant. (2020) 26:835–44. doi: 10.1016/j.bbmt.2019.08.029 PMC7060124 31505228

[16] Fisher SA Cutler A Doree C Brunskill SJ Stanworth SJ Navarrete C. Mesenchymal stromal cells as treatment or prophylaxis for acute or chronic graft-versus-host disease in haematopoietic stem cell transplant (HSCT) recipients with a haematological condition. Cochrane Database Syst Rev. (2019) 1:Cd009768. doi: 10.1002/14651858.CD009768.pub2 30697701 PMC6353308

[17] Garcia-Olmo D Gilaberte I Binek M D´Hoore AJL Lindner D Selvaggi F. Follow-up study to evaluate the long-term safety and efficacy of darvadstrocel (Mesenchymal stem cell treatment) in patients with perianal fistulizing crohn’s disease: ADMIRE-CD phase 3 randomized controlled trial. Dis Colon Rectum. (2022) 65:713–20. doi: 10.1097/DCR.0000000000002325 PMC8985696 34890373

[18] Petrou P Kassis I Levin N Paul F Backner Y Benoliel T. Beneficial effects of autologous mesenchymal stem cell transplantation in active progressive multiple sclerosis. Brain. (2020) 143:3574–88. doi: 10.1093/brain/awaa333 33253391

[19] Sarsenova M Issabekova A Abisheva S Rutskaya-Moroshan K Ogay V Saparov A. Mesenchymal stem cell-based therapy for rheumatoid arthritis. Int J Mol Sci. (2021) 22:11592. doi: 10.3390/ijms222111592 34769021 PMC8584240

[20] Galipeau J Sensébé L. Mesenchymal stromal cells: clinical challenges and therapeutic opportunities. Cell Stem Cell. (2018) 22:824–33. doi: 10.1016/j.stem.2018.05.004 PMC6434696 29859173

[21] Kurtzberg J Abdel-Azim H Carpenter P Chaudhury S Horn B Mahadeo K. A phase 3, single-arm, prospective study of remestemcel-L, ex vivo culture-expanded adult human mesenchymal stromal cells for the treatment of pediatric patients who failed to respond to steroid treatment for acute graft-versus-host disease. Biol Blood Marrow Transplant. (2020) 26:845–54. doi: 10.1016/j.bbmt.2020.01.018 PMC8322819 32018062

[22] Zhao K Lin R Fan Z Chen X Wang Y Huang F. Mesenchymal stromal cells plus basiliximab, calcineurin inhibitor as treatment of steroid-resistant acute graft-versus-host disease: a multicenter, randomized, phase 3, open-label trial. J Hematol Oncol. (2022) 15:22. doi: 10.1186/s13045-022-01240-4 35255929 PMC8900437

[23] Mullard A. 2024 FDA approvals. Nat Rev Drug Discovery. (2025). doi: 10.1038/d41573-025-00001-5 39747473

[24] Su Y Xu C Cheng W Zhao Y Sui L Zhao Y. Pretreated mesenchymal stem cells and their secretome: enhanced immunotherapeutic strategies. Int J Mol Sci. (2023) 24:1277. doi: 10.3390/ijms24021277 36674790 PMC9864323

[25] Li J Wu Z Zhao L Liu Y Su Y Gong X. The heterogeneity of mesenchymal stem cells: an important issue to be addressed in cell therapy. Stem Cell Res Ther. (2023) 14:381. doi: 10.1186/s13287-023-03587-y 38124129 PMC10734083

[26] Blanco B Herrero-Sánchez MD Rodríguez-Serrano C García-Martínez ML Blanco JF Muntión S. Immunomodulatory effects of bone marrow versus adipose tissue-derived mesenchymal stromal cells on NK cells: implications in the transplantation setting. Eur J Haematol. (2016) 97:528–37. doi: 10.1111/ejh.2016.97.issue-6 27118602

[27] Shi Y Su J Roberts AI Shou P Rabson AB Ren G. How mesenchymal stem cells interact with tissue immune responses. Trends Immunol. (2012) 33:136–43. doi: 10.1016/j.it.2011.11.004 PMC3412175 22227317

[28] Najar M Krayem M Merimi M Burny A Meuleman N Bron D. Insights into inflammatory priming of mesenchymal stromal cells: functional biological impacts. Inflammation Res. (2018) 67:467–77. doi: 10.1007/s00011-018-1131-1 29362849

[29] Lacey DC Simmons PJ Graves SE Hamilton JA. Proinflammatory cytokines inhibit osteogenic differentiation from stem cells: implications for bone repair during inflammation. Osteoarthritis Cartilage. (2009) 17:735–42. doi: 10.1016/j.joca.2008.11.011 19136283

[30] Sullivan CB Porter RM Evans CH Ritter T Shaw G Barry F. TNFα and IL-1β influence the differentiation and migration of murine MSCs independently of the NF-κB pathway. Stem Cell Res Ther. (2014) 5:104. doi: 10.1186/scrt492 25163844 PMC4177434

[31] Wang M Crisostomo PR Herring C Meldrum KK Meldrum DR. Human progenitor cells from bone marrow or adipose tissue produce VEGF, HGF, and IGF-I in response to TNF by a p38 MAPK-dependent mechanism. Am J Physiol Regul Integr Comp Physiol. (2006) 291:R880–4. doi: 10.1152/ajpregu.00280.2006 16728464

[32] Duijvestein M Wildenberg ME Welling MM Hennink S Molendijk I van Zuylen VL. Pretreatment with interferon-γ Enhances the therapeutic activity of mesenchymal stromal cells in animal models of colitis. Stem Cells. (2011) 29:1549–58. doi: 10.1002/stem.698 21898680

[33] Herger N Heggli I Mengis T Devan J Arpesella L Brunner F. Impacts of priming on distinct immunosuppressive mechanisms of mesenchymal stromal cells under translationally relevant conditions. Stem Cell Res Ther. (2024) 15:65. doi: 10.1186/s13287-024-03677-5 38443999 PMC10916130

[34] Gómez-Ferrer M Amaro-Prellezo E Dorronsoro A Sánchez-Sánchez R Vicente Á Cosín-Roger J. HIF-overexpression and pro-inflammatory priming in human mesenchymal stromal cells improves the healing properties of extracellular vesicles in experimental crohn’s disease. Int J Mol Sci. (2021) 22:11269. doi: 10.3390/ijms222011269 34681929 PMC8540690

[35] Pfeffer LM. The role of nuclear factor κB in the interferon response. J Interferon Cytokine Res. (2011) 31:553–9. doi: 10.1089/jir.2011.0028 PMC3128784 21631354

[36] Schütze S Wiegmann K Machleidt T Krönke M. TNF-induced activation of NF-kappa B. Immunobiology. (1995) 193:193–203. doi: 10.1016/S0171-2985(11)80543-7 8530143

[37] Hayden MS Ghosh S. Regulation of NF-κB by TNF family cytokines. Semin Immunol. (2014) 26:253–66. doi: 10.1016/j.smim.2014.05.004 PMC4156877 24958609

[38] Sabio G Davis RJ. TNF and MAP kinase signalling pathways. Semin Immunol. (2014) 26:237–45. doi: 10.1016/j.smim.2014.02.009 PMC4099309 24647229

[39] Valledor AF Arpa L Sánchez-Tilló E Comalada M Casals C Xaus J. IFN-γ–mediated inhibition of MAPK phosphatase expression results in prolonged MAPK activity in response to M-CSF and inhibition of proliferation. Blood. (2008) 112:3274–82. doi: 10.1182/blood-2007-11-123604 18682602

[40] Yang HT Cohen P Rousseau S. IL-1beta-stimulated activation of ERK1/2 and p38alpha MAPK mediates the transcriptional up-regulation of IL-6, IL-8 and GRO-alpha in HeLa cells. Cell Signal. (2008) 20:375–80. doi: 10.1016/j.cellsig.2007.10.025 18065201

[41] Pahl HL. Activators and target genes of Rel/NF-kappaB transcription factors. Oncogene. (1999) 18:6853–66. doi: 10.1038/sj.onc.1203239 10602461

[42] Liu T Zhang L Joo D Sun SC. NF-κB signaling in inflammation. Signal Transduct Target Ther. (2017) 2:17023–. doi: 10.1038/sigtrans.2017.23 PMC5661633 29158945

[43] Cicchese JM Evans S Hult C Joslyn LR Wessler T Millar JA. Dynamic balance of pro- and anti-inflammatory signals controls disease and limits pathology. Immunol Rev. (2018) 285:147–67. doi: 10.1111/imr.2018.285.issue-1 PMC6292442 30129209

[44] Al-Qahtani AA Alhamlan FS Al-Qahtani AA. Pro-inflammatory and anti-inflammatory interleukins in infectious diseases: A comprehensive review. Trop Med Infect Dis. (2024) 9:13. doi: 10.3390/tropicalmed9010013 38251210 PMC10818686

[45] Kim EY Moudgil KD. Regulation of autoimmune inflammation by pro-inflammatory cytokines. Immunol Lett. (2008) 120:1–5. doi: 10.1016/j.imlet.2008.07.008 18694783 PMC2577081

[46] Fernández-Santos ME Garcia-Arranz M Andreu EJ García-Hernández AM López-Parra M Villarón E. Optimization of mesenchymal stromal cell (MSC) manufacturing processes for a better therapeutic outcome. Front Immunol. (2022) 13:918565. doi: 10.3389/fimmu.2022.918565 35812460 PMC9261977

[47] Gao F Chiu SM Motan DA Zhang Z Chen L Ji HL. Mesenchymal stem cells and immunomodulation: current status and future prospects. Cell Death Dis. (2016) 7:e2062. doi: 10.1038/cddis.2015.327 26794657 PMC4816164

[48] Zhou Y Yamamoto Y Xiao Z Ochiya T. The immunomodulatory functions of mesenchymal stromal/stem cells mediated via paracrine activity. J Clin Med. (2019) 8:1025. doi: 10.3390/jcm8071025 31336889 PMC6678920

[49] Zhou Y Tsai TL Li WJ. Strategies to retain properties of bone marrow-derived mesenchymal stem cells ex vivo. Ann N Y Acad Sci. (2017) 1409:3–17. doi: 10.1111/nyas.2017.1409.issue-1 28984359 PMC5730506

[50] Di Nicola M Carlo-Stella C Magni M Milanesi M Longoni PD Matteucci P. Human bone marrow stromal cells suppress T-lymphocyte proliferation induced by cellular or nonspecific mitogenic stimuli. Blood. (2002) 99:3838–43. doi: 10.1182/blood.V99.10.3838 11986244

[51] Haddad R Saldanha-Araujo F. Mechanisms of T-cell immunosuppression by mesenchymal stromal cells: what do we know so far? BioMed Res Int. (2014) 2014:216806. doi: 10.1155/2014/216806 25025040 PMC4082893

[52] Duffy MM Ritter T Ceredig R Griffin MD. Mesenchymal stem cell effects on T-cell effector pathways. Stem Cell Res Ther. (2011) 2:34. doi: 10.1186/scrt75 21861858 PMC3219065

[53] Chinnadurai R Copland IB Patel SR Galipeau J. IDO-independent suppression of T cell effector function by IFN-γ-licensed human mesenchymal stromal cells. J Immunol. (2014) 192:1491–501. doi: 10.4049/jimmunol.1301828 24403533

[54] Lee BC Kang KS. Functional enhancement strategies for immunomodulation of mesenchymal stem cells and their therapeutic application. Stem Cell Res Ther. (2020) 11:397. doi: 10.1186/s13287-020-01920-3 32928306 PMC7491075

[55] Wang Q Yang Q Wang Z Tong H Ma L Zhang Y. Comparative analysis of human mesenchymal stem cells from fetal-bone marrow, adipose tissue, and Warton’s jelly as sources of cell immunomodulatory therapy. Hum Vaccines Immunotherapeut. (2016) 12:85–96. doi: 10.1080/21645515.2015.1030549 PMC4962749 26186552

[56] Abbasi B Shamsasenjan K Ahmadi M Beheshti SA Saleh M. Mesenchymal stem cells and natural killer cells interaction mechanisms and potential clinical applications. Stem Cell Res Ther. (2022) 13:97. doi: 10.1186/s13287-022-02777-4 35255980 PMC8900412

[57] Spaggiari GM Capobianco A Becchetti S Mingari MC Moretta L. Mesenchymal stem cell-natural killer cell interactions: evidence that activated NK cells are capable of killing MSCs, whereas MSCs can inhibit IL-2-induced NK-cell proliferation. Blood. (2006) 107:1484–90. doi: 10.1182/blood-2005-07-2775 16239427

[58] Maqbool M Algraittee SJR Boroojerdi MH Sarmadi VH John CM Vidyadaran S. Human mesenchymal stem cells inhibit the differentiation and effector functions of monocytes. Innate Immun. (2020) 26:424–34. doi: 10.1177/1753425919899132 PMC7903531 32635840

[59] Nauta AJ Kruisselbrink AB Lurvink E Willemze R Fibbe WE. Mesenchymal stem cells inhibit generation and function of both CD34+-derived and monocyte-derived dendritic cells. J Immunol. (2006) 177:2080–7. doi: 10.4049/jimmunol.177.4.2080 16887966

[60] Han X Ding S Jiang H Liu G. Roles of macrophages in the development and treatment of gut inflammation. Front Cell Dev Biol. (2021) 9. doi: 10.3389/fcell.2021.625423 PMC7960654 33738283

[61] Ahamada MM Jia Y Wu X. Macrophage polarization and plasticity in systemic lupus erythematosus. Front Immunol. (2021) 12. doi: 10.3389/fimmu.2021.734008 PMC8721097 34987500

[62] Krzyszczyk P Schloss R Palmer A Berthiaume F. The role of macrophages in acute and chronic wound healing and interventions to promote pro-wound healing phenotypes. Front Physiol. (2018) 9:419. doi: 10.3389/fphys.2018.00419 29765329 PMC5938667

[63] Lu D Xu Y Liu Q Zhang Q. Mesenchymal stem cell-macrophage crosstalk and maintenance of inflammatory microenvironment homeostasis. Front Cell Dev Biol. (2021) 9:681171. doi: 10.3389/fcell.2021.681171 34249933 PMC8267370

[64] Lu D Jiao X Jiang W Yang L Gong Q Wang X. Mesenchymal stem cells influence monocyte/macrophage phenotype: Regulatory mode and potential clinical applications. Biomed Pharmacother. (2023) 165:115042. doi: 10.1016/j.biopha.2023.115042 37379639

[65] Wang J Liu Y Ding H Shi X Ren H. Mesenchymal stem cell-secreted prostaglandin E2 ameliorates acute liver failure via attenuation of cell death and regulation of macrophage polarization. Stem Cell Res Ther. (2021) 12:15. doi: 10.1186/s13287-020-02070-2 33413632 PMC7792134

[66] Liao Y Li G Zhang X Huang W Xie D Dai G. Cardiac nestin(+) mesenchymal stromal cells enhance healing of ischemic heart through periostin-mediated M2 macrophage polarization. Mol Ther. (2020) 28:855–73. doi: 10.1016/j.ymthe.2020.01.011 PMC7054724 31991111

[67] Li Y Zhang D Xu L Dong L Zheng J Lin Y. Cell-cell contact with proinflammatory macrophages enhances the immunotherapeutic effect of mesenchymal stem cells in two abortion models. Cell Mol Immunol. (2019) 16:908–20. doi: 10.1038/s41423-019-0204-6 PMC6884632 30778166

[68] Philipp D Suhr L Wahlers T Choi Y-H Paunel-Görgülü A. Preconditioning of bone marrow-derived mesenchymal stem cells highly strengthens their potential to promote IL-6-dependent M2b polarization. Stem Cell Res Ther. (2018) 9:286. doi: 10.1186/s13287-018-1039-2 30359316 PMC6202843

[69] Liu C Xu Y Lu Y Du P Li X Wang C. Mesenchymal stromal cells pretreated with proinflammatory cytokines enhance skin wound healing via IL-6-dependent M2 polarization. Stem Cell Res Ther. (2022) 13:414. doi: 10.1186/s13287-022-02934-9 35964139 PMC9375394

[70] Dave M Dev A Somoza RA Zhao N Viswanath S Mina PR. MSCs mediate long-term efficacy in a Crohn’s disease model by sustained anti-inflammatory macrophage programming via efferocytosis. NPJ Regener Med. (2024) 9:6. doi: 10.1038/s41536-024-00347-1 PMC10799947 38245543

[71] Jerkic M Szaszi K Laffey JG Rotstein O Zhang H. Key role of mesenchymal stromal cell interaction with macrophages in promoting repair of lung injury. Int J Mol Sci. (2023) 24:3376. doi: 10.3390/ijms24043376 36834784 PMC9965074

[72] Vázquez A Fernández-Sevilla LM Jiménez E Pérez-Cabrera D Yañez R Subiza JL. Involvement of mesenchymal stem cells in oral mucosal bacterial immunotherapy. Front Immunol. (2020) 11:567391. doi: 10.3389/fimmu.2020.567391 33329530 PMC7711618

[73] Liu GY Liu Y Lu Y Qin YR Di GH Lei YH. Short-term memory of danger signals or environmental stimuli in mesenchymal stem cells: implications for therapeutic potential. Cell Mol Immunol. (2016) 13:369–78. doi: 10.1038/cmi.2015.11 PMC4856802 25942600

