# Supplementary figures and images for: Improving the therapeutic profile of MSCs: Cytokine priming reduces donor-dependent heterogeneity and enhances their immunomodulatory capacity
Source: Front Immunol. 2025 Feb 17;16:1473788. doi: 10.3389/fimmu.2025.1473788 (PMC11872697; doi:10.3389/fimmu.2025.1473788)

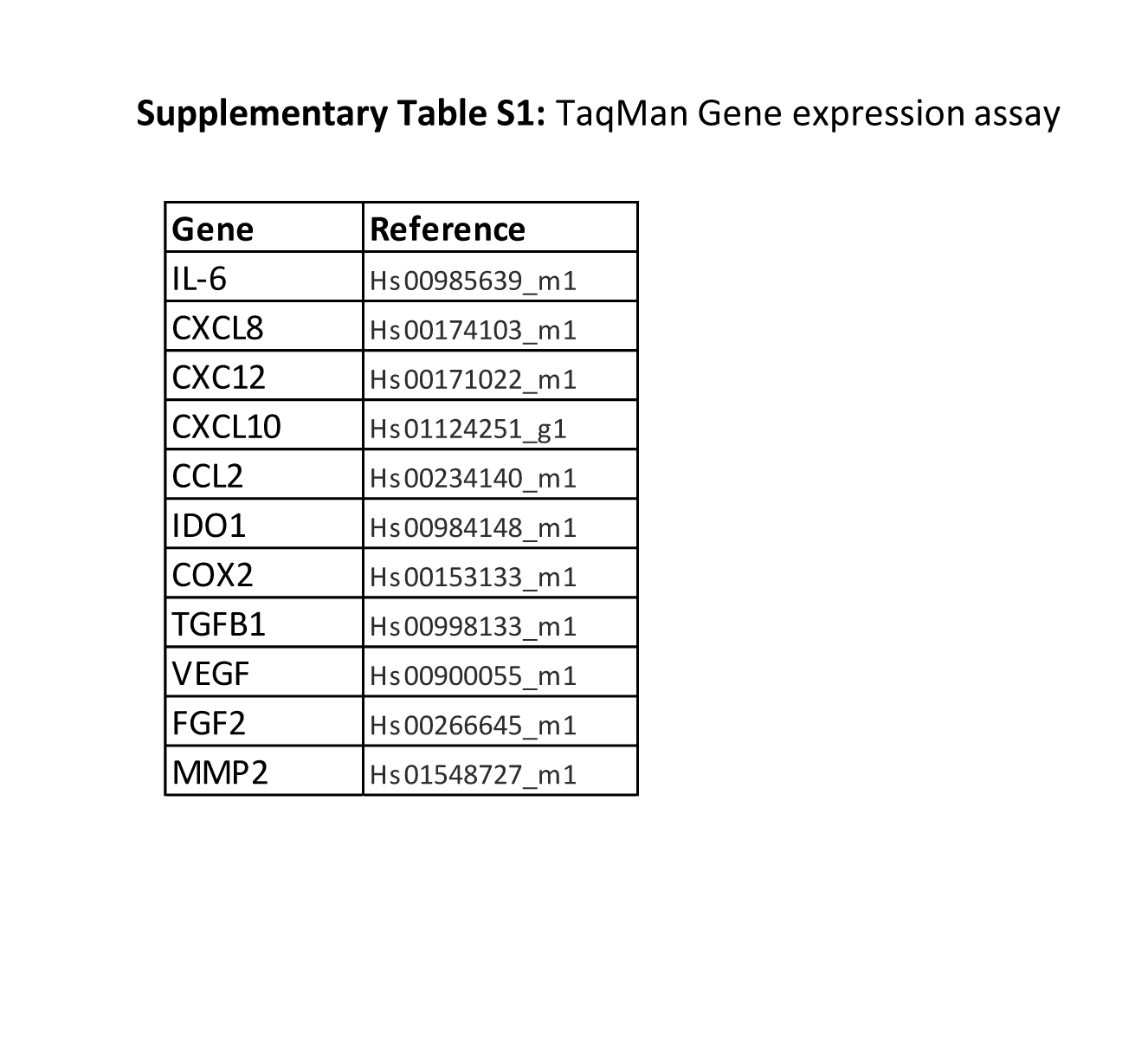

Supplement: Supplementary file 1 [file DataSheet1.zip › Supplementary Table S1.TIF]

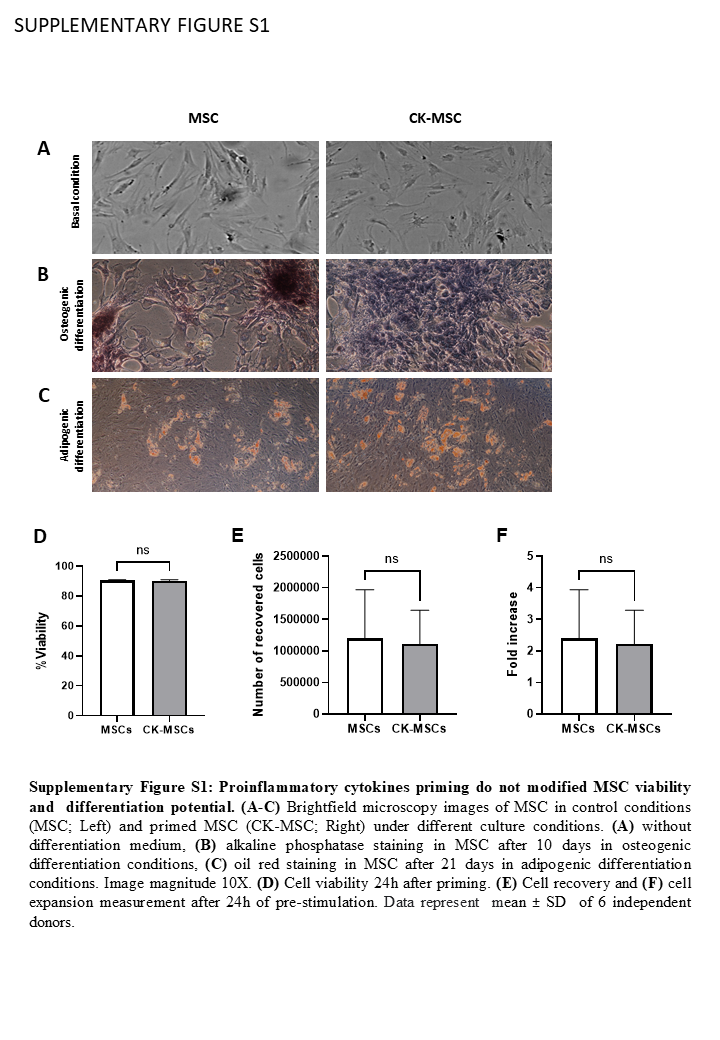

Supplement: Supplementary file 1 [file DataSheet1.zip › Supplementary Figure S1.tif]

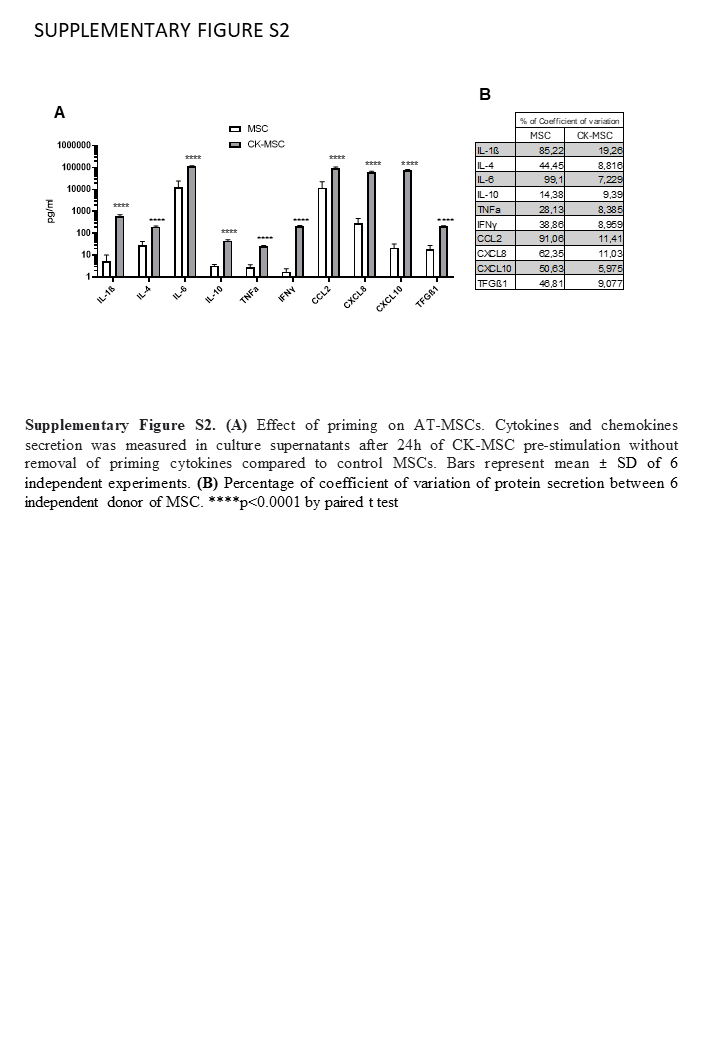

Supplement: Supplementary file 1 [file DataSheet1.zip › Supplementary Figure S2.tif]
